# Sample Size for Successful Genome-Wide Association Study of Major Depressive Disorder

**DOI:** 10.3389/fgene.2018.00227

**Published:** 2018-06-28

**Authors:** Jo Nishino, Hidenori Ochi, Yuta Kochi, Tatsuhiko Tsunoda, Shigeyuki Matsui

**Affiliations:** ^1^Department of Medical Science Mathematics, Medical Research Institute, Tokyo Medical and Dental University, Tokyo, Japan; ^2^CREST, JST, Tokyo, Japan; ^3^Division of Frontier Medical Science, Programs for Biomedical Research Graduate School of Biomedical Science, Department of Gastroenterology and Metabolism, Hiroshima University, Hiroshima, Japan; ^4^Laboratory for Digestive Diseases, RIKEN Center for Integrative Medical Sciences, Hiroshima, Japan; ^5^Laboratory for Autoimmune Diseases, RIKEN Center for Integrative Medical Sciences, Yokohama, Japan; ^6^Laboratory for Medical Science Mathematics, RIKEN Center for Integrative Medical Sciences, Yokohama, Japan; ^7^Risk Analysis Research Center, The Institute of Statistical Mathematics, Tachikawa, Tokyo, Japan; ^8^Department of Biostatistics, Nagoya University Graduate School of Medicine, Nagoya, Japan

**Keywords:** major depressive disorder, genome-wide association studies (GWAS), semi-parametric hierarchical mixture model (SP-HMM), effect-size distribution, genome-wide significance, sample size

## Abstract

Major depressive disorder (MDD) is a complex, heritable psychiatric disorder. Advanced statistical genetics for genome-wide association studies (GWASs) have suggested that the heritability of MDD is largely explained by common single nucleotide polymorphisms (SNPs). However, until recently, there has been little success in identifying MDD-associated SNPs. Here, based on an empirical Bayes estimation of a semi-parametric hierarchical mixture model using summary statistics from GWASs, we show that MDD has a distinctive polygenic architecture consisting of a relatively small number of risk variants (~17%), e.g., compared to schizophrenia (~42%). In addition, these risk variants were estimated to have very small effects (genotypic odds ratio ≤ 1.04 under the additive model). Based on the estimated architecture, the required sample size for detecting significant SNPs in a future GWAS was predicted to be exceptionally large. It is noteworthy that the number of genome-wide significant MDD-associated SNPs would rapidly increase when collecting 50,000 or more MDD-cases (and the same number of controls); it can reach as much as 100 SNPs out of nearly independent (linkage disequilibrium pruned) 100,000 SNPs for ~120,000 MDD-cases.

## Introduction

Major depressive disorder (MDD) is a common, complex disorder with a high lifetime prevalence of ~15% (Kessler et al., 2003) and a moderate heritability of 31–42% (Sullivan et al., 2000). Etiological understanding of MDD is potentially of great impact on individuals and public health. Several statistical genetics approaches have suggested that a large portion of the heritability of MDD is explained by common single nucleotide polymorphisms (SNPs) (Lubke et al., 2012; Lee et al., 2013). However, no significant MDD-associated variant has been discovered even in a large genome-wide association study (GWAS) with around 9,500 cases by the Psychiatric Genomics Consortium (PGC) (Major Depressive Disorder Working Group of the Psychiatric Genomics Consortium, 2013; Levinson et al., 2014). Most recently, two studies have respectively identified one genome-wide significant SNP for particular subpopulations with relatively less phenotypic heterogeneity. One used severe Han Chinese women patients (Cai et al., 2015) and the other reanalyzed the data collected from the PGC with stratification by self-reported age (Power et al., 2017). In contrast, as a GWAS analysis for a general population without restriction to particular subpopulations, Hyde et al. (2016) used European self-reported phenotyped data from a consumer genomics company, 23andMe, composed of a massive sample size of 75,607 cases and 231,747 controls, and identified 15 independent loci associated with major depression. However, one possible limitation of this study is the validity of self-reported phenotype information. Therefore, although it provided a candidate list of disease-associated loci for the first time, further GWASs are warranted for discovery of new variants associated with MDD.

The power to discover new disease-associated variants critically depends on the underlying genetic architecture, i.e., the number of risk loci and their frequencies and effect sizes. One possible reason for the difficulty in identifying variants associated with MDD might relate to the disease's high prevalence/low heritability feature. Based on these perspectives, Wray et al. (2012) carefully quantified that sample sizes 4 to 5-fold greater are needed for GWASs of MDD compared with schizophrenia (SCZ), assuming the same number and frequency of risk variants underlying SCZ and MDD.

In this study, utilizing GWAS summary data of PGC (Major Depressive Disorder Working Group of the Psychiatric Genomics Consortium, 2013), we unbiasedly estimated the proportion of disease-associated variants and their effect size distribution with the use of our recently developed empirical Bayes method with a semi-parametric hierarchical mixture model (SP-HMM) (Nishino et al., 2018). Based on the estimated genetic architectures by this method, we explain why GWASs of MDD have failed to discover disease-associated variants, through comparisons with other diseases, including SCZ (Ripke et al., 2014), type 2 diabetes (T2D) (Morris et al., 2012) with similar heritability and prevalence to MDD, and Crohn's disease (CD) (Liu et al., 2015), for which GWASs to date have successfully identified disease-associated variants. We also analyzed GWAS data for other psychiatric disorders including autism spectrum disorders (ASDs) (Autism Spectrum Disorder Working Group of the Psychiatry Genomics Consortium, 2015) and anorexia nervosa (AN) (Boraska et al., 2014), which have not had much progress in GWAS. We then predicted a curve of the number of significant SNPs or the number of new discoveries for various sizes of future GWASs. This prediction would be particularly useful for designing future GWASs for complex diseases for which limited disease-associated variants have been identified.

## Results

### Proportion of disease-associated SNPs and their effect-size distributions

**Figure 1:**
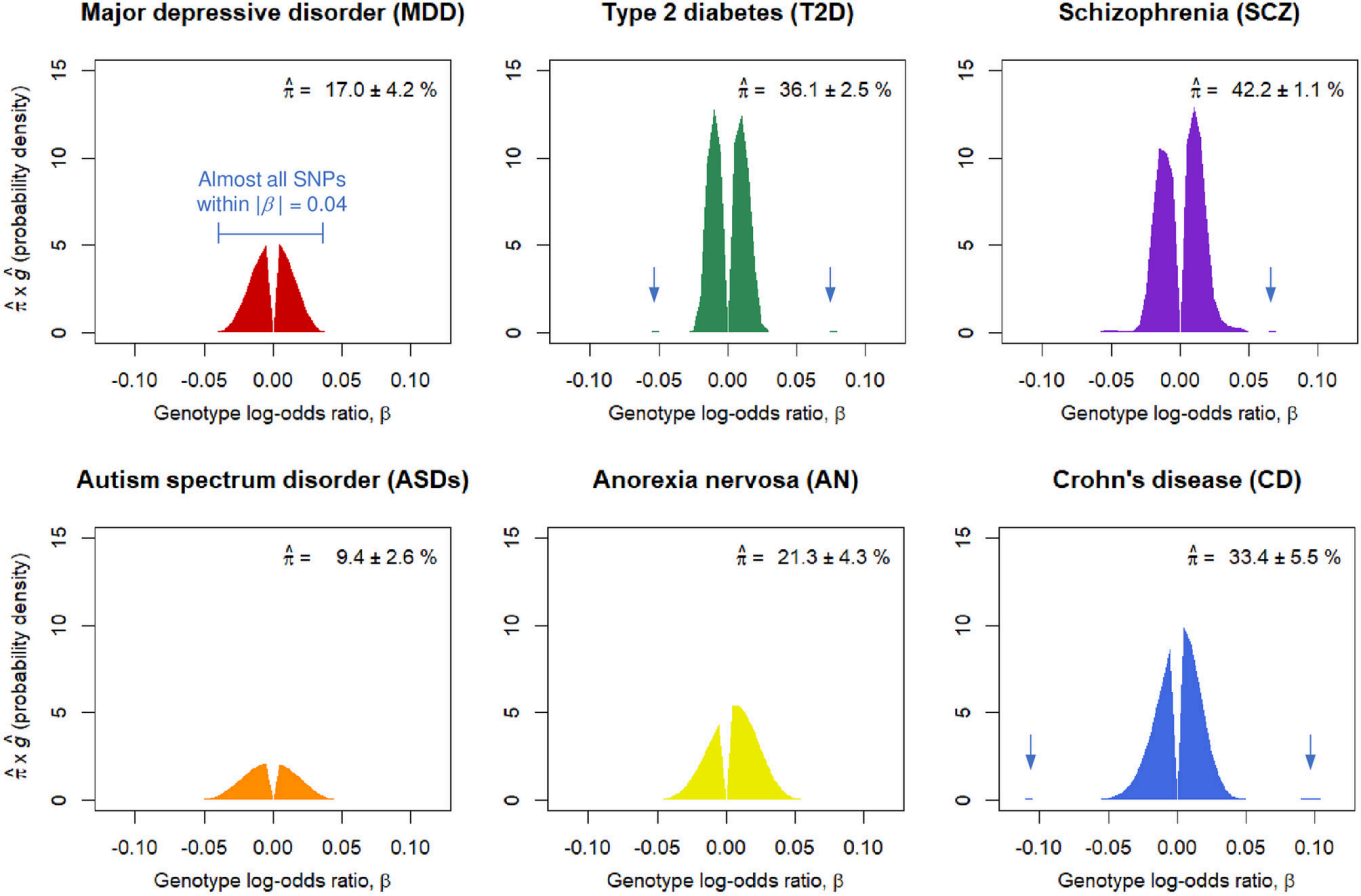
Estimated proportions of disease-associated SNPs, $\hat{\pi }$, and effect-size distributions for disease-associated SNPs, ĝ. $\hat{\pi }$ corresponds to the areas under the curves. Numbers after the plus-minus signs (“±”) are standard errors by 100 parametric bootstrap samples based on the estimated SP-HMM. Vertical allows in the figures indicate small peaks with relatively large effects.

We obtained nearly independent pruned SNP sets consisting of around *m* = 100,000 SNPs for the six GWASs (Table S1). The SP-HMM was fitted to each pruned SNP set to estimate the proportion of disease-associated SNPs, π, and their effect size distribution, *g*, non-parametrically (Figure 1). The proportion of disease-associated SNPs, π, for SCZ was estimated to be the largest ($\hat{\pi }\;\sim \;42.2\;\%$), i.e., SCZ was highly polygenic, followed by T2D and CD. ASDs was the least polygenic ($\hat{\pi }\;\sim \;9.4\;\%$) among the six GWASs. MDD was the second least polygenic, $\hat{\pi }\;\sim \;17.0\;\%$. For AN, π was estimated to be intermediate, $\hat{\pi }\;\sim \;21.3\;\%$.

Non-parametric estimation of *g* flexibly characterized the effect-size distributions for the six diseases as follows. A noteworthy feature in the effect-size distribution of disease-associated SNPs, *g*, for MDD is that there were few SNPs with large effects; most were within |β| = 0.03 (genotypic odds ratio = 1.03 under the additive model) and almost all SNPs were within |β| = 0.04 (odds ratio = 1.04). For ASDs, effect sizes were estimated to be relatively small among the six GWASs; almost all SNPs were within |β| = 0.05. For CD, we had many disease-associated SNPs with effect sizes near or more than |β| = 0.05 or odds ratio = 1.05, and also peaks of effects around |β| = 0.1. The estimated distribution of *g* for SCZ lay mostly within a range of |β| ≤ 0.03, but with peaks at relatively large effects of |β| 0.05 or larger. AN had relatively large effects, particularly in the positive signed region. For T2D, while most disease-associated SNPs were within |β| = 0.03, there was a small portion of disease-associated SNPs with the effect sizes near or more than |β| = 0.05.

### Prediction of the number of significant SNPs

**Figure 2:**
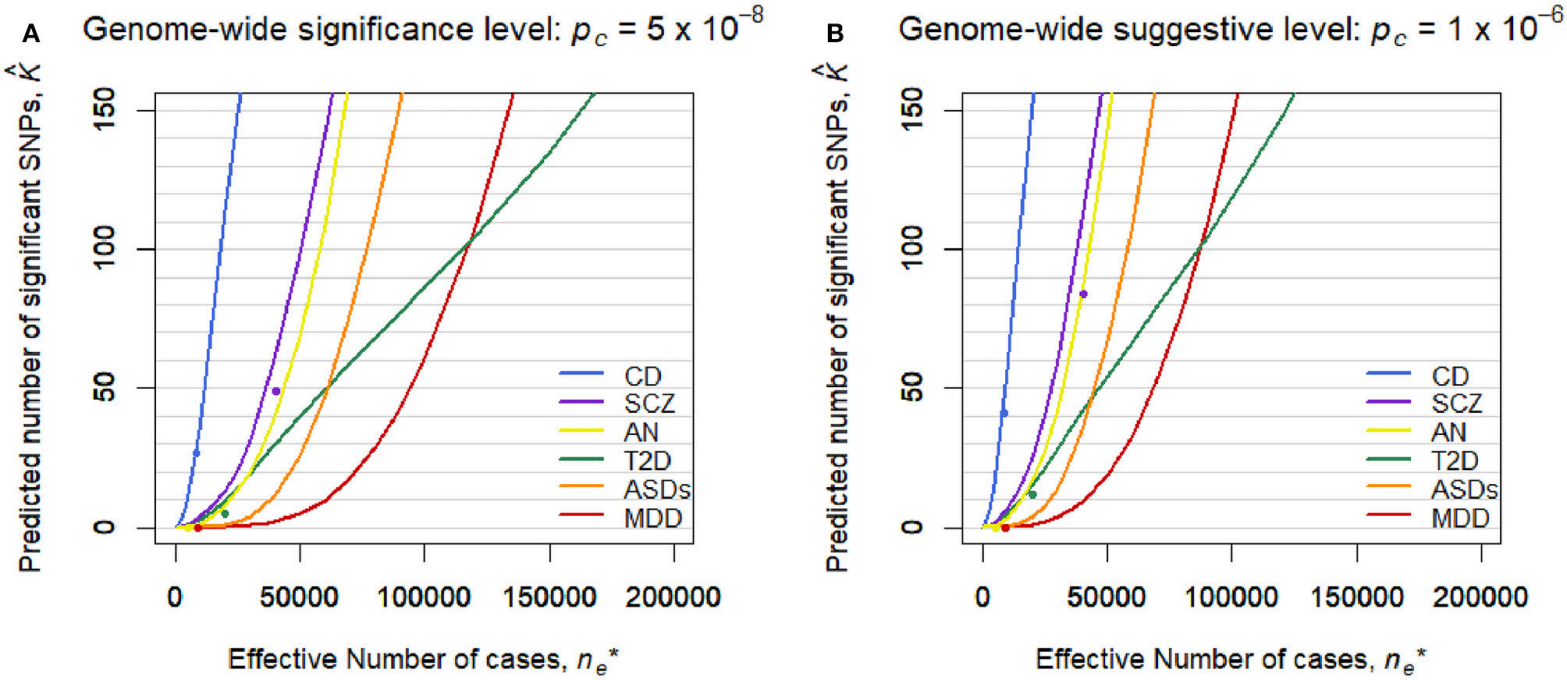
Predicted number of significant SNPs, $\hat{K}$, under the estimated SP-HMM. Predicted number of significant SNPs, $\hat{K}$, was calculated assuming m^*^ = 100,000 independent SNPs in the “future” GWASs. Dots show observed values in the pruned SNP sets of current GWAS data. **(A)** Genome-wide significance level: *p*_*c*_ = 5 × 10^−8^. **(B)** Genome-wide suggestive level: *p*_*c*_ = 10^−6^.

Figure 2 shows the predicted number of significant SNPs, $\hat{K}$, with the genome-wide significance level of *p*_*c*_ = 5 × 10^−8^ (Figure 2A) and suggestive level of *p*_*c*_ = 1 × 10^−6^ (Figure 2B) for each disease, assuming m^*^ = 100,000 independent SNPs in a future GWAS. Also, Figure S1 shows $\hat{K}$ with 95% confidence intervals for each disease in log scale. We first confirmed that the observed number of significant SNPs in the pruned SNP sets in the current GWASs, shown in dots, was well-captured by the predicted curves in all the diseases. In both levels of the statistical significance thresholds, the number of significant SNPs was predicted to be by far the largest for CD in all ranges of the effective number of cases. The predicted number of statistical significance was the second largest for SCZ. Those for AN were next to and near those for SCZ. For detecting 1, 10, and 100 genome-wide significant SCZ-associated SNPs, 7,000, 18,000, and 51,000 effective number of cases was predicted to be needed, respectively. We observed that, for MDD, the predicted number of statistically significant SNPs was exceptionally small in both levels of the statistical significance thresholds (Figure 2). Nevertheless, the predicted number for MDD rapidly increases when $n_{e}^{*}$ > 50,000. For detecting 1, 10, and 100 genome-wide significant MDD-associated SNPs, 34,000, 61,000, and 118,000 effective number of cases was predicted to be needed, respectively (Figure S2). For detecting 1, 10, and 100 genome-wide significant SCZ-associated SNPs, 7,000, 18,000, and 51,000 effective number of cases was predicted to be needed (Figure S2), which was 4.9, 3.4, and 2.3 times larger than those for SCZ, respectively. Those numbers were 4.9, 3.4, and 2.3 times larger than those for SCZ, respectively. For ASDs, the predicted curves of the number of disease-associated SNPs with significance in both levels of statistical significance thresholds lay in the middle of those for SCZ and MDD (Figure 2). For T2D, in case of $n_{e}^{*}$ < 2,000, the number of detected SNPs was predicted to be close to those for SCZ and AN. However, as the sample size increased, the predicted detections for T2D with the genome-wide significance and suggestive level became smaller than those for ASDs, or even for MDD.

## Discussion

Although GWASs have played a critical role in discovering disease-associated variants for many complex diseases, this approach has not necessarily worked well for some diseases, including psychiatric disorders such as MDD. In this paper, we have attempted to explain the reason for the failure in GWASs for such diseases, through estimating the genetic architecture based on an empirical Bayes estimation of a flexible, semi-parametric hierarchical mixture model (Nishino et al., 2018) using summary data from the existing GWASs (Figure 1).

For the six diseases examined, we commonly observed that the genetic basis consisted of enormous variants, ranging from $\hat{\pi }\;\sim$ 9.4 to 42.2% in the nearly independent 100,000 genome-wide SNPs, with small effects (majority of genotypic odds ratio for risk alleles are within 1.05 under the additive model). In regard to MDD, the SP-HMM clarified the distinctive feature of polygenicity; the proportion of MDD-associated SNPs was relatively small, $\hat{\pi }\;\sim \;17.0\;\%$ compared with other diseases (SCZ, T2D, CD, ASDs, and AN), and the absolute effect sizes for almost all of the non-null SNPs were very small, |β| ≤ 0.04, in the pruned GWAS data from PGC (Major Depressive Disorder Working Group of the Psychiatric Genomics Consortium, 2013) (Figure 1).

However, this difficulty in discovering MDD-associated variants can be addressed with increased sample sizes. A prediction on the number of discoveries in a future GWAS based on the estimated genetic architecture indicated that the number of significant SNPs can substantially increase when collecting 50,000 or more MDD-cases (and the same number of controls). It can reach as much as 100 SNPs out of 100,000 independent SNPs for ~120,000 MDD-cases (Figure 2 and Figure S2). Note that the results cannot rule out the importance of taking into account rare variants, environment-gene interaction (Caspi et al., 2010), and heterogeneity possibly resolved by stratified analysis (Power et al., 2017).

One reviewer of this article kindly informed us that the MDD-PGC group identified 44 independent significant SNPs using the seven cohorts (130,664 cases and 330,470 controls in total) including PGC data with 16,823 cases and 25,632 controls (Wray et al., 2018). One part of the results of that study seems to be consistent with our estimate that the effect sizes of MDD-associated SNPs were very small, i.e., |β| ≤ 0.04 (Figure 1); the crude odds ratio estimates of 41 SNPs of 44 significant SNPs in the PGC (2017) were 0.96, 0.97, 1.03, or 1.04, that is $|\hat{\beta }|\leq 0.04$ (Table 1 in Wray et al., 2018). Note that the true effect sizes of the 44 SNPs would be generally smaller than those of estimates, as the crude estimate is subject to the “winners curse.” On the other hand, by the present method, the number of significant SNPs assuming 100,000 independent SNPs was predicted as 355.1 (95% confidence interval 239.7–683.3) using the sample with 130,664 cases and 330,470 controls, which largely exceeded the observed number, 44 (Figure S1). (Note that our estimation targeting 100,000 independent SNPs is supposed to underestimate the number of significant SNPs seen in practical situations, where SNPs with higher association (e.g., lower *P*-value) are preferentially selected among “all SNPs” so that linkage disequilibrium (LD) among selected SNPs are nearly independent). The discrepancy between our prediction and the observation could be due to the difference between the PGC cohort data and data from the other six cohorts, especially, self-reported data from 23andMe with 130,664 cases and 330,470 controls, which accounted for the large proportion of the total cohorts. In fact, the SNP heritability estimates in observed scales were much smaller for 23andMe data (0.038) than for PGC data (0.128) (Hyde et al., 2016). Our over prediction suggests that for MDD, possibly for other diseases, phenotyping methods have great impact on the number of significant SNPs. Despite the reduced power, self-reported data from a consumer genomics company, e.g., 23andMe, would increase in importance due to its utility. It is our intention to clarify the difference in effect-size of disease-associated variants between self-reported data and established phenotyped data.

In addition to MDD, the prediction analysis can be used for comparing the number of discoveries among diseases. For example, the number of future discoveries for AN is expected to be of the same extent as for SCZ, while the number for ASDs is predicted to be intermediate between those for SCZ and MDD.

Using a method similar to the present study, Park et al. (2010) investigated the relationship between sample size and the number of significant disease-associated SNPs based on the estimated effect size distribution of disease-associated SNPs. This method, however, is limited to relatively large effect sizes in the effect-size estimation due to the need to use SNPs with some significant level, and requires adjustment for the winner's curse (selection bias in using top significant SNPs) in the estimation. Stahl et al. (2012) proposed a method to estimate the proportion of disease-associated SNPs and the effect-size distribution using an approximate Bayesian polygenic analysis (ABPA). The application to evaluate the relationship between sample size and the number of significant disease-associated SNPs has been limited to few studies because of technical complexity and excess computational burden with many simulations (to our knowledge, Ripke et al., 2013 applied the ABPA method). There are also several “Gaussian mixture models” to estimate the underlying effect sizes using the z-scores for SNPs as the inputs (Thompson et al., 2015; Holland et al., 2016). These models are applicable to investigate the relationship between sample size and the number of significant disease-associated SNPs, although the authors did not directly study this problem. Note that the definitions of effect sizes in the above existing methods are different from that of the SP-HMM, e.g., 2*f*(1−*f*)β^2^for Park et al. (2010), and $\sqrt{2f\left(1-f\right)}\beta$ for Thompson et al. (2015), where *f* is the allele frequency.

The features of the SP-HMM make it quick and easy to compute the number of significant disease-associated SNPs given sample sizes understanding the estimated proportion of the disease-associated SNPs and effect-size distribution where the effect size is easy to understand, defined as the genotype log-odds ratio under the additive model, β. In making inference about a SNP regarding its null/non-null association with disease status, the number of components, in principle, is two (i.e., null and non-null components). In modeling the non-null component (effect size distribution), the parametric approach, e.g., finite normal mixture models with several components, is a popular choice. Unlike such a parametric model, we assume a non-parametric distribution as a “single” non-null component to cover all such non-null components. This is the interpretation for the modeling formula given in the subsection “Semi-parametric Hierarchical Mixture Model (SP-HMM)” in the Materials and Methods section. Meanwhile, in estimation using the expectation–maximization (EM) algorithm we can see our model as that with “so many” non-null components (the number of components = *B*, described in the subsection “Semi-parametric Hierarchical Mixture Model (SP-HMM)” in the discretized effect size distribution used in the estimation algorithm). We have shown that with 3–5,000 or more cases (and the same number of controls), the estimates of π and *g* are fairly accurate, leading to reliable estimates of the number of significant disease-associated SNPs (Nishino et al., 2018). Note that our prediction of the number of significant SNPs targets “the LD-pruned SNP set” in the future GWAS data, where SNPs would be randomly selected so that LDs among SNPs should be *r*^2^ < 0.1. This limitation regarding the target SNPs (i.e., the LD-pruned SNP set) will be addressed in future work. Although we assumed 100,000 SNPs in the LD-pruned set from the observations in Table S1, a different number of SNPs in the LD-pruned set would be considered in the proposed approach. This is because the number should depend on the effective size of study population, as is the case for “the effective number of chromosome segments” (*M*_*e*_; the key determinant of the accuracy of genomic prediction) does, i.e., *M*_*e*_ = 2.938 $N_{e}^{0.965}$ under 30 Morgan in total, where *N*_*e*_ is the effective population size (Lee et al., 2017).

In conclusion, our prediction analysis is generally useful for designing future GWASs for complex diseases, through estimating additional number of cases (and controls) needed to be collected in a single cohort study, or additional cohorts (sample sets) needed to be included in a meta-analysis, and for discovering a given number of new disease-associated variants.

## Materials and methods

### Semi-parametric hierarchical mixture model (SP-HMM)

To estimate polygenic architectures of the six diseases, we used the SP-HMM (Nishino et al., 2018). The SP-HMM estimates the proportion of disease-associated SNPs, π, and their effect size distribution, *g*, non-parametrically, using GWAS summary statistics on effect sizes (genotype log-odds ratios) which often are available through Web sites. The “non-parametric estimation of *g*” enables us to flexibly characterize the effect-size distributions without any assumptions for forms of the distribution. The SP-HMM assumes independence among SNPs, as was justified by pruning SNP described below. The SP-HMM has been validated through various types of polygenic scenarios and the required sample size was confirmed to be around 3–5,000 or more (see Nishino et al., 2018 for more details about the SP-HMM). The SP-HMM is briefly described in the following.

Letting *a* and *A* be the derived and ancestral alleles status, respectively. The genotypes *AA*, *Aa*, and *aa* in each SNP assumed to have dosages *x*_*j*_ = 0, 1, and 2, respectively. Under the additive allele dosage model, we defined the effect size, β_*j*_, as the genotype log-odds ratio for the *j*-th SNP of the total *m* SNPs. The estimate of β_*j*_ was denoted by $Y_{j}={\hat{\beta }}_{j}.$ For *Y*_*j*_'s, a two-component mixture model with null and non-null SNPs components is assumed:

$$\begin{array}{l} f_{j}\left(y_{j}\right)=\left(1-\pi \right)f_{0j}\left(y_{j}\right)\;+\;\pi f_{1j}\left(y_{j}\right), \end{array}$$

where *f*_0*j*_ and *f*_1*j*_ are the probability densities for null and non-null SNPs, respectively, and π is the probability of being non-null. Let ${\hat{V}}_{{\hat{\beta }}_{j}}$ be an empirical variance estimate of ${\hat{\beta }}_{j}$. Asymptotic distribution of ${\hat{\beta }}_{j}$ were assumed. For null SNPs, we specified $y_{j}\;\sim \;f_{0j}\left(y_{j}\right)=N\left(0,{\hat{V}}_{{\hat{\beta }}_{j}}\right)$. For non-null SNPs, we assumed the hierarchical structure: $y_{j}|\beta_{j}\;\sim \;f_{1j}\left(y_{j}|\beta_{j}\right)=N\left(\beta_{j},{\hat{V}}_{{\hat{\beta }}_{j}}\right)$ and β_*j*_ ~ *g*, where the effect-size distribution *g* is unspecified. We regard this model as a semi-parametric model, as the standard asymptotic normality is assumed for ${\hat{\beta }}_{j}$ at the individual SNP level, while its true effect size β_*j*_ follows a non-parametric prior distribution *g*. The assumption of independence among *y*_*j*_'s would be reasonable for a set of LD-pruned SNPs (for the details about pruning see the subsection of “GWAS Data”). We estimated the priors, π and *g*, based on the data by applying an expectation–maximization (EM) algorithm, that is, empirical Bayes estimation. The non-parametric estimate of *g* was discrete with mass points ***p*** = (*p*_1_*, p*_2_*, …, p*_*B*_) at a series of nonzero points ***b*** = (*b*_1_*, b*_2_*, …, b*_*B*_) (*b*_1_ < *b*_2_ < ··· < *b*_*B*_). We set *b*_1_ = −0.3 and *b*_*B*_ = 0.3 (0.74 and 1.35 in odds ratio). The number grid point *B* = 120 was used, such that ***b*** = (−0.300, −0.295, …, −0.005, 0.005, …, 0.295, 0.300). The initial value of π and the initial distribution of *g* were important and determined by a careful procedure (for details, see Nishino et al., 2018). To estimate standard errors of $\hat{\pi }$ and 95% confidence interval of $\hat{K}$, the parametric bootstrap method based on the estimated SP-HMM was used. The validity of the estimation using the SP-HMM has been demonstrated via an extensive simulation experiment under various scenarios in terms of sample size, π, *g*, and possible correlations among SNP (Nishino et al., 2018).

### Prediction of the number of significant SNPs

For the *j*-th SNP, the power to detect an association with effect size β_*j*_, *Power*_*j*_(β_*j*_), is given by

$$\begin{array}{l} Power_{j}\left(\beta_{j}\right)=\Phi_{\beta_{j}/\sqrt{{\hat{V}}_{{\hat{\beta }}_{j}}},1}\left(-z_{c}\right)+\left(1-\Phi_{\beta_{j}/\sqrt{{\hat{V}}_{{\hat{\beta }}_{j}}},1}\left(z_{c}\right)\right), \end{array}$$

where Φ_μ, 1_(·) denotes the cumulative distribution function of the normal distribution with mean μ and unit variance, and *z*_*c*_ denotes the rejection threshold determined by a significance level, *p*_*c*_, satisfying $z_{c}=\Phi_{0,1}^{-1}\left(1-p_{c}/2\right)$). In this study, *p*_*c*_ = 5 × 10^−8^ (the genome-wide “significant” threshold) and *p*_*c*_ = 1 × 10^−6^ (the genome-wide “suggestive” threshold) were used. Under the SP-HMM, the rejection probability, i.e., the probability that the *j*-th SNP is significant, is given by

(1) $$\begin{array}{l} P_{j}=\left(1-\pi \right)Power_{j}\left(0\right)+\pi \int_{-\infty }^{\infty }Power_{j}\left(\beta_{j}\right)g\left(\beta_{j}\right)d\beta_{j}. \end{array}$$

Let *n*_*r*_ and *n*_*s*_ be the sample sizes for cases and controls, respectively, in an existing GWAS from which we can estimate the SP-HMM. In addition, we envisage a “future” GWAS with $n_{r}^{*}$ cases and $n_{s}^{*}$ controls. Based on the formula (1), the probability of significance for the *j*-th SNP in the future GWAS can be obtained through replacing ${\hat{V}}_{{\hat{\beta }}_{j}}$ with ${\hat{V}}_{{\hat{\beta }}_{j}}\times$1/(1/*n*_*r*_ + 1/*n*_*s*_) × (1/$n_{r}^{*}$ + 1/$n_{s}^{*}$), since the empirical variance of ${\hat{\beta }}_{j}$ is approximately proportional to the sum of inverses of case and control sample sizes. This approximation has been used in the GWAS meta-analysis (Willer et al., 2010). The derivation in the logistic regression for “large sample and small effect-size” limit was done elsewhere (e.g., by Lin and Sullivan, 2009). The number of significant SNPs, *K*, in the future data set consisting of m^*^ SNPs is then predicted as

(2) $$\begin{array}{l} \hat{K}=m^{*}\times \bar{P}. \end{array}$$

where $\bar{P}$ is the average rejection probability over all SNPs in the SNP set, $\bar{P}=\overset{m}{\underset{j=1}{\sum }}P_{j}/m$, replacing ${\hat{V}}_{{\hat{\beta }}_{j}}$ with ${\hat{V}}_{{\hat{\beta }}_{j}}\times$1/(1/*n*_*r*_ + 1/*n*_*s*_) × (1/$n_{r}^{*}$ + 1/$n_{s}^{*}$), π with $\hat{\pi }$ and *g* with ĝ, respectively, in the formula (1). We set m^*^ = 100,000 for targeting 100,000 pruned SNPs. Since the term (1/$n_{r}^{*}$ + 1/$n_{s}^{*}$) determines the predicted number of significant SNPs, $\hat{K}$, we define the “effective number of cases” as $n_{e}^{*}=2/\left(1/n_{r}^{*}+1/n_{s}^{*}\right)$. As such, we can obtain a curve of the number of significant SNPs in a future GWAS, $\hat{K}$, as a function of its sample size, $n_{e}^{*}$, based on the estimated SP-HMM using the existing GWAS data.

### GWAS data

The six sets of GWAS summary statistics for MDD (Major Depressive Disorder Working Group of the Psychiatric Genomics Consortium, 2013), SCZ (Ripke et al., 2014), T2D (Morris et al., 2012), CD (Liu et al., 2015), ASDs (Autism Spectrum Disorder Working Group of the Psychiatry Genomics Consortium), and AN (Boraska et al., 2014) were used, which are all available online (MDD, SCZ, ASDs and AN, www.med.unc.edu/pgc/downloads; T2D, http://www.diagram-consortium.org/; IBD, http://www.ibdgenetics.org/downloads.html; see Table S1 for sample size). To restrict analysis to well-imputed, high-quality variants, we used only SNPs that existed on the HapMap 3 reference panel (International HapMap 3 Consortium., 2010). For the pruned SNP sets, we included SNPs randomly, irrespective of degrees of association such that no SNPs in the set were in *r*^2^ > 0.1, as done in the previous work (Nishino et al., 2018). We selected one SNP randomly from all the SNP data and SNPs in LD (*r*^2^ > 0.1) with the selected SNP removed. This was repeated until no SNPs remained. LD information(*r*^2^) was extracted from the HapMap database (HapMap phases I+II+III, release 27) (International HapMap 3 Consortium., 2010). With this pruning process, we could interpret the significant SNPs as SNPs linked to independent causal variants. Meanwhile, the SP-HMM analysis evaluates the marginal effect of the pruned SNPs and underestimates the effects of causal variants; estimated effect-size distributions should be smaller than those of causal variants, and the estimates $\hat{\pi }$ × (the number of SNPs in the SNP sets) would give conservative estimates of the number of causal variants. Nevertheless, the SP-HMM estimation reflects the effects of the causal variants for each disease through linkage disequilibrium. LD information was retrieved from the HapMap (International HapMap 3 Consortium., 2010) data base (HapMap phases I+II+III, release 27). The ancestral/derived alleles for each SNP were determined from dbSNP (Nishino et al., 2018). We calculated the estimate of log-odds ratio for the *j*-th SNP, ${\hat{\beta }}_{j}$, and its variance, ${\hat{V}}_{{\hat{\beta }}_{j}}$ for applying the SP-HMM to the pruned SNP sets and predicting of number of significant SNPs.

### Empirical validation for prediction of the number of significant SNPs

We validated our approach for predicting the number of significant SNPs using hypothetical “current” and “future” GWAS data; we fitted the SP-HMM to the “current” GWAS data with smaller sample size to predict the number of significant SNPs in the “future” GWAS data with larger sample size, and we compared the predicted value with the observed one. The three pairs of GWAS summary statistics for SCZ (for “current” data, Cross-Disorder Group of the Psychiatric Genomics Consortium, 2013; for “future” data, Major Depressive Disorder Working Group of the Psychiatric GWAS Consortium, 2013), bipolar disorder (for 'current' data, Cross-Disorder Group of the Psychiatric Genomics Consortium, 2013; for 'future' data, Ripke et al., 2014), and coronary artery disease (for “current” data, The Coronary Artery Disease (C4D) Genetics Consortium, 2011; for “future” data, Schunkert et al., 2011) are all available online (SCZ, bipolar disorder, www.med.unc.edu/pgc/downloads; coronary artery disease, www.cardiogramplusc4d.org/data-downloads/). The quality control and pruning for the SNP data were done as described in the previous subsection, “GWAS Data.” For SCZ, bipolar disorder, and coronary artery disease, there were 101314, 96681, and 79512 SNPs in the pruned sets, respectively. Those values were set as m^*^ in the formula (2). The number of SNPs was smaller for coronary artery disease (79512), as the original GWAS summary data have been imputed using HapMap data. Table S2 shows the validation results. The observed number of significant SNPs for each disease was well-predicted by our approach.

## Author contributions

JN and SM: Conceptualization; JN: Formal analysis; TT and SM: Funding acquisition; JN and SM: Writing original draft; HO, YK and TT: Writing review and editing.

### Conflict of interest statement

The authors declare that the research was conducted in the absence of any commercial or financial relationships that could be construed as a potential conflict of interest.

## References

[B1] Autism Spectrum Disorder Working Group of the Psychiatry Genomics Consortium (2015). Dataset: PGC-ASD Summary Statistics From a Meta-Analysis of 5,305 ASD-Diagnosed Cases and 5,305 Pseudocontrols of European Descent (Based on Similarity to CEPH Reference Genotypes). Available online at: http://www.med.unc.edu/pgc/results-anddownloads.

[B2] Boraska V. Franklin C. S. Floyd J. A. Thornton L. M. Huckins L. M. Southam L.. (2014). A genome-wide association study of anorexia nervosa. Mol. Psychiatry 19, 1085–1094. 10.1038/mp.2013.187 24514567 PMC4325090

[B3] Cai N. Bigdeli T. B. Kretzschmar W. Li Y. H. Liang J. Q. Song L. (2015). Sparse whole-genome sequencing identifies two loci for major depressive disorder. Nature 523, 588–591. 10.1038/nature14659 26176920 PMC4522619

[B4] Caspi A. Sugden K. Moffitt T. E. Taylor A. Craig I. W. Harrington H.. (2010). Influence of life stress on depression: moderation by a polymorphism in the 5-HTT gene. Science 301, 386–389. 10.1126/science.1083968 12869766

[B5] Coronary Artery Disease (C4D) Genetics Consortium Consortium. (2011). A genome-wide association study in Europeans and South Asians identifies five new loci for coronary artery disease. Nat. Genet. 43, 339–344. 10.1038/ng.782 21378988

[B6] Cross-Disorder Group of the Psychiatric Genomics Consortium (2013). Identification of risk loci with shared effects on five major psychiatric disorders: a genome-wide analysis. Lancet 381, 1371–1379. 10.1016/S0140-6736(12)62129-1 23453885 PMC3714010

[B7] Holland D. Wang Y. Thompson W. K. Schork A. Chen C. H. Lo M. T.. (2016). Estimating effect sizes and expected replication probabilities from GWAS summary statistics. Front. Genet. 7:15. 10.3389/fgene.2016.00015 26909100 PMC4754432

[B8] Hyde C. L. Nagle M. W. Tian C. Chen X. Paciga S. A. Wendland J. R.. (2016). Identification of 15 genetic loci associated with risk of major depression in individuals of European descent. Nat. Genet. 48, 1031–1036. 10.1038/ng.3623 27479909 PMC5706769

[B9] International HapMap 3 Consortium (2010). Integrating common and rare genetic variation in diverse human populations. Nature 467, 52–58. 10.1038/nature09298 20811451 PMC3173859

[B10] Kessler R. Berglund P. Demler O. Jin R. Koretz D. Merikangas K. (2003). The epidemiology of major depressive disorder. J. Am. Med. Assoc. 23, 3095–3105. 10.1001/jama.289.23.3095 12813115

[B11] Lee S. H. Clark S. Van Der Werf J. H. J. (2017). Estimation of genomic prediction accuracy from reference populations with varying degrees of relationship. PLoS ONE 12:e0189775. 10.1371/journal.pone.0189775 29267328 PMC5739427

[B12] Lee S. H. Ripke S. Neale B. M. Faraone S. V. Purcell S. M. Perlis R. H.. (2013). Genetic relationship between five psychiatric disorders estimated from genome-wide SNPs. Nat. Genet. 45, 984–994. 10.1038/ng.2711 23933821 PMC3800159

[B13] Levinson D. F. Mostafavi S. Milaneschi Y. Rivera M. Ripke S. Wray N. R.. (2014). Genetic studies of major depressive disorder: why are there no genome-wide association study findings and what can we do about it? Biol. Psychiatry 76, 510–512. 10.1016/j.biopsych.2014.07.029 25201436 PMC4740915

[B14] Lin D. Y. Sullivan P. F. (2009). Meta-analysis of genome-wide association studies with overlapping subjects. Am. J. Hum. Genet. 85, 862–872. 10.1016/j.ajhg.2009.11.001 20004761 PMC2790578

[B15] Liu J. Z. van Sommeren S. Huang H. Ng S. C. Alberts R. Takahashi A.. (2015). Association analyses identify 38 susceptibility loci for inflammatory bowel disease and highlight shared genetic risk across populations. Nat. Genet. 47, 979–989. 10.1038/ng.3359 26192919 PMC4881818

[B16] Lubke G. H. Hottenga J. J. Walters R. Laurin C. De Geus E. J. C. Willemsen G.. (2012). Estimating the genetic variance of major depressive disorder due to all single nucleotide polymorphisms. Biol. Psychiatry 72, 707–709. 10.1016/j.biopsych.2012.03.011 22520966 PMC3404250

[B17] Major Depressive Disorder Working Group of the Psychiatric Genomics Consortium (2013). A mega-analysis of genome-wide association studies for major depressive disorder. Mol. Psychiatry 18, 497–511. 10.1038/mp.2012.21 22472876 PMC3837431

[B18] Morris A. P. Voight B. F. Teslovich T. M. Ferreira T. Segrè A. V. Steinthorsdottir V.. (2012). Large-scale association analysis provides insights into the genetic architecture and pathophysiology of type 2 diabetes. Nat. Genet. 44, 981–990. 10.1038/ng.2383 22885922 PMC3442244

[B19] Nishino J. Kochi Y. Shigemizu D. Kato M. Ikari K. Ochi H.. (2018). Empirical Bayes estimation of semi-parametric hierarchical mixture models for unbiased characterization of polygenic disease architectures. Front. Genet. 9:115. 10.3389/fgene.2018.00115 29740473 PMC5928254

[B20] Park J.-H. H. Wacholder S. Gail M. H. Peters U. Jacobs K. B. Chanock S. J.. (2010). Estimation of effect size distribution from genome-wide association studies and implications for future discoveries. Nat. Genet. 42, 570–575. 10.1038/ng.610 20562874 PMC4615599

[B21] Power R. A. Tansey K. E. Buttenschon H. N. Cohen-Woods S. Bigdeli T. Hall L. S.. (2017). Genome-wide association for major depression through age at onset stratification: major depressive disorder working group of the psychiatric genomics consortium. Biol. Psychiatry 81, 325–335. 10.1016/j.biopsych.2016.05.010 27519822 PMC5262436

[B22] Ripke S. Neale B. M. Corvin A. Walters J. T. R. Farh K.-H. Holmans P. A. (2014). Biological insights from 108 schizophrenia-associated genetic loci. Nature 511, 421–427. 10.1038/nature13595 25056061 PMC4112379

[B23] Ripke S. O'Dushlaine C. Chambert K. Moran J. L. Kahler A. K. Akterin S.. (2013). Genome-wide association analysis identifies 13 new risk loci for schizophrenia. Nat. Genet. 45, 1150–1159. 10.1038/ng.2742 23974872 PMC3827979

[B24] Schunkert H. Konig I. R. Kathiresan S. Reilly M. P. Assimes T. L. Holm H.. (2011). Large-scale association analysis identifies 13 new susceptibility loci for coronary artery disease. Nat. Genet. 43, 333–338. 10.1038/ng.784 21378990 PMC3119261

[B25] Stahl E. A. Wegmann D. Trynka G. Gutierrez-Achury J. Do R. Voight B. F.. (2012). Bayesian inference analyses of the polygenic architecture of rheumatoid arthritis. Nat. Genet. 44, 483–489. 10.1038/ng.2232 22446960 PMC6560362

[B26] Sullivan P. F. Neale M. C. Kendler K. S. (2000). Genetic epidemiology of major depression : review and meta-analysis. Am. J. Psychiatry 157, 1552–1562. 10.1176/appi.ajp.157.10.1552 11007705

[B27] Thompson W. K. Wang Y. Schork A. J. Witoelar A. Zuber V. Xu S.. (2015). An empirical Bayes mixture model for effect size distributions in genome-wide association studies. PLoS Genet. 11:e1005717. 10.1371/journal.pgen.1005717 26714184 PMC5456456

[B28] Willer C. J. Li Y. Abecasis G. R. (2010). METAL: fast and efficient meta-analysis of genomewide association scans. Bioinformatics 26, 2190–2191. 10.1093/bioinformatics/btq340 20616382 PMC2922887

[B29] Wray N. R. Pergadia M. L. Blackwood D. H. R. Penninx B. W. J. H. Gordon S. D. Nyholt D. R.. (2012). Genome-wide association study of major depressive disorder: new results, meta-analysis, and lessons learned. Mol. Psychiatry 17, 36–48. 10.1038/mp.2010.109 21042317 PMC3252611

[B30] Wray N. R. Ripke S. Mattheisen M. Trzaskowski M. Byrne E. M. Abdellaoui A.. (2018). Genome-wide association analyses identify 44 risk variants and refine the genetic architecture of major depression. Nat. Genet. 50, 668–681. 10.1038/s41588-018-0090-3 29700475 PMC5934326

