# Supplementary material for: Sample Size for Successful Genome-Wide Association Study of Major Depressive Disorder
Source: Front Genet. 2018 Jun 28;9:227. doi: 10.3389/fgene.2018.00227 (PMC6032046; doi:10.3389/fgene.2018.00227)
Supplement: Supplementary file 1 [file Presentation_1.pdf]

Table S1. GWAS data used in this study.

	Cases	Controls	Number of the pruned SNPs
Major depressive disorder (MDD)	9,240	9,519	104,000
Autism spectrum disorder (ASDs)	5,305	5,305	108,000
Type 2 diabetes (T2D)	12,171	56,862	99,000
Anorexia Nervosa (AN)	3,495	10,982	105,000
Schizophrenia (SCZ)	35,476	46,389	100,000
Crohn's disease (CD)	5,956	15,927	107,000

Table S2. Validation for predicting the number of significant SNPs by real GWAS data.

Disease	Effective number of cases (# of cases: # of controls)		Significance level	Number of significant SNPs in 'future' GWAS	
	'Current' GWAS	'Future' GWAS		Prediction [95% CI]	Observed
Bipolar disorder	5,706 (4,820:6,990)	8,742 (9,250:7,481)	5×10^{-8}	0.17 [0.07, 1.54]	0
			1×10^{-6}	1.28 [0.70, 4.13]	1
Coronary artery disease	15,239 (15,062:15,420)	33,101 (64,762:22,233)	5×10^{-8}	4.67 [0.77, 13.22]	3
			1×10^{-6}	7.77 [3.22, 19.11]	6
Schizophrenia	8,478 (7,736:9,379)	40,205 (46,389:35,476)	5×10^{-8}	54.36 [30.99, 104.19]	48
			1×10^{-6}	127.71 [85.74, 199.88]	88

Figure S1. Predicted number of significant SNPs, $\hat{K}$, with their confidence interval under the estimated SP-HMM.

a

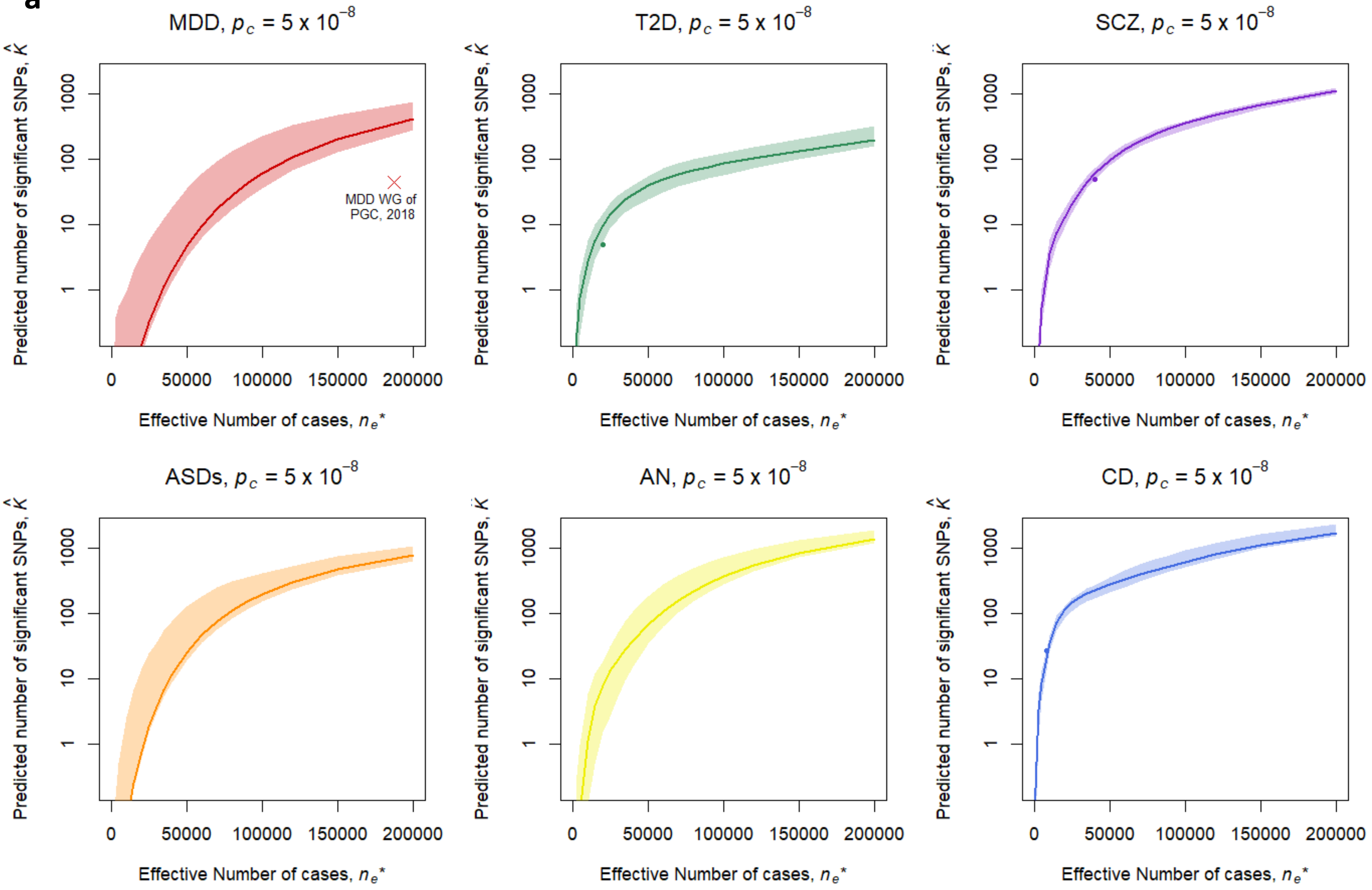

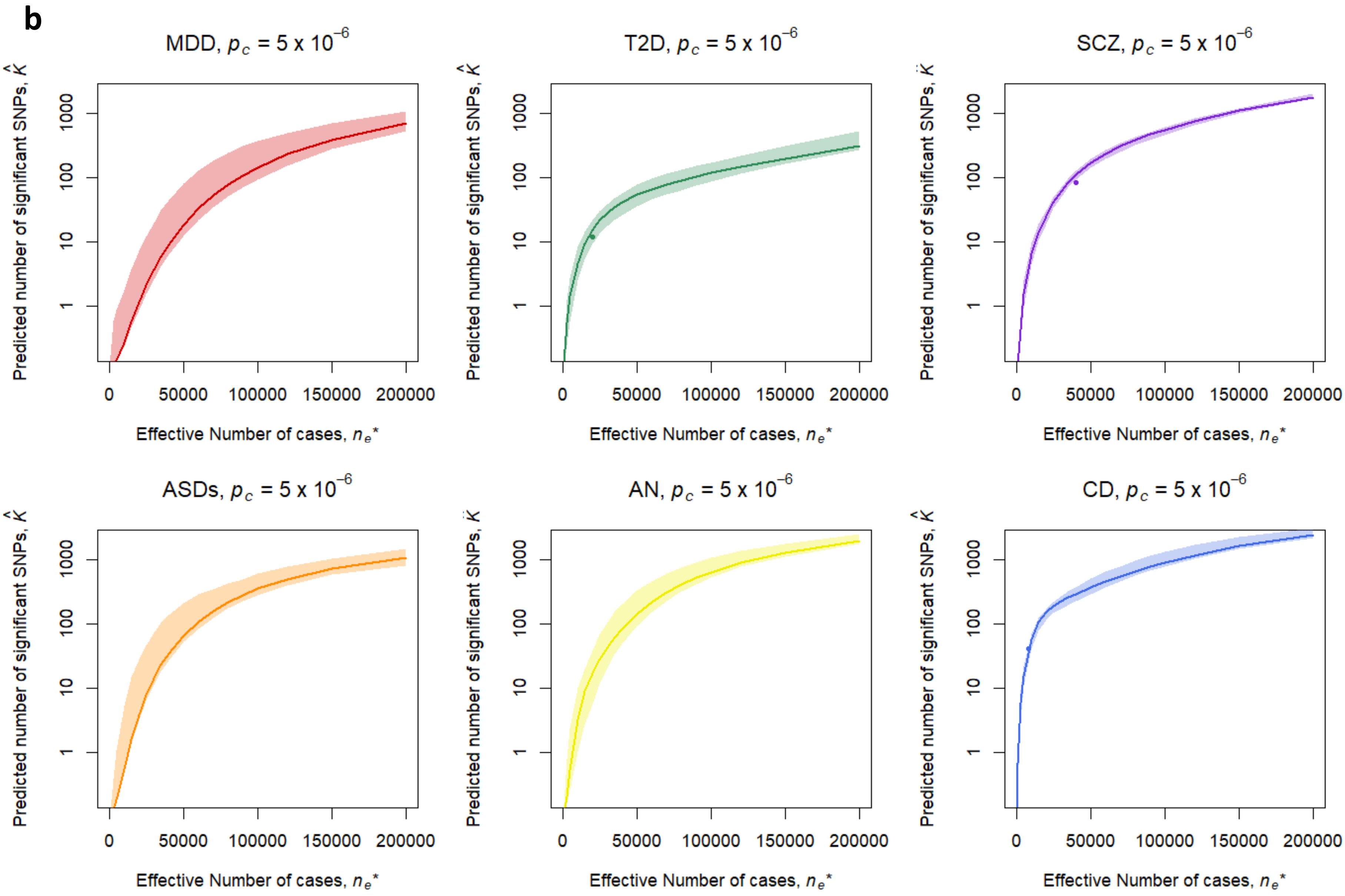
b
MDD, p_c = 5 x 10^{-6}
T2D, p_c = 5 x 10^{-6}
SCZ, p_c = 5 x 10^{-6}
ASDs, p_c = 5 x 10^{-6}
AN, p_c = 5 x 10^{-6}
CD, p_c = 5 x 10^{-6}
Predicted number of significant SNPs, $\hat{K}$
1
10
100
1000
0
50000
100000
150000
200000
Effective Number of cases, n_e*

Figure S1.

Predicted number of significant SNPs, $\widehat{K}$, was calculated assuming m^* = 100,000 independent SNPs in the 'future' GWASs (lines). Shared regions are 95% confidence intervals, by 100 parametric bootstrap samples based on the estimated SP-HMM. Dots show observed values in the pruned SNP sets of current GWAS data. 'MDD WG of PGC, 2018': Observed number of significant SNPs by Major Depressive Disorder Working Group of the Psychiatric Genomics Consortium (2018). (a) Genome-wide significance level: $p_c = 5 \times 10^{-8}$. (b) Genome-wide suggestive level: $p_c = 10^{-6}$.

Figure S2. Predicted effective number of cases (n_e^*) required for getting 1, 10, and 100 significant SNPs, $\hat{K}$, in the 'future' GWASs.

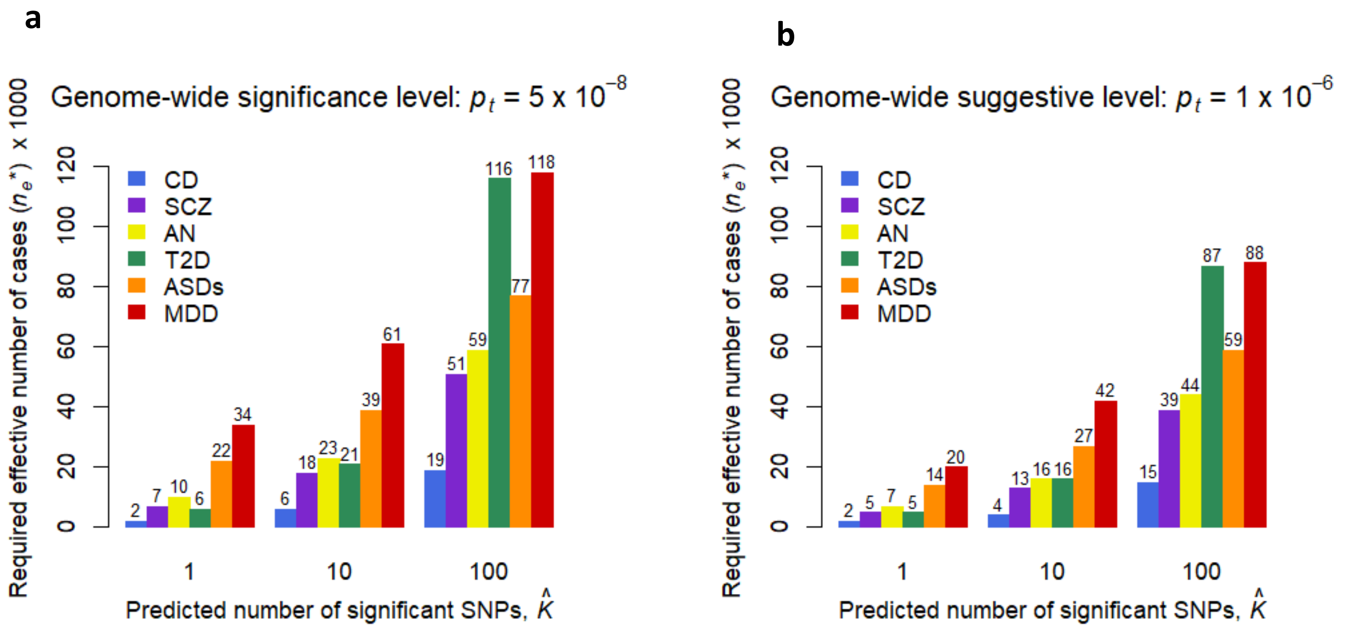

The estimates for given number of significant SNPs was obtained based on the formula (2) by the gird search by increasing n_e^* from 1,000–200,000 by increments of 1,000. m^*= 100,000 independent SNPs were assumed. (a) Genome-wide significance level: p_c = 5×10^{-8}. (b) Genome-wide suggestive level: $p_c = 10^{-6}$.
